# Post-castration variations in weight gain in a cohort of young adult male cats*

**DOI:** 10.1017/jns.2014.37

**Published:** 2014-09-30

**Authors:** Alfreda Wei, Andrea J. Fascetti, Kyoungmi Kim, Jon J. Ramsey

**Affiliations:** 1 VM – Molecular Biosciences, University of California, Davis, CA 95616, USA; 2 Department of Public Health Sciences, School of Medicine, University of California, Davis, CA 95616, USA

**Keywords:** Obesity, Castration, Cats, BW, body weight

## Abstract

The predisposition of cats to gain weight following neutering is well established; however, there is little information about the distribution and range of post-neutering weight gains observed in cats under a controlled environment. This retrospective study investigated 6-month post-castration weight gain and distribution of percentage body weight (BW) change in a cohort of twenty, male domestic shorthair cats relative to a control group of intact cats. Cats were matched in age (2·0–2·6 years), husbandry conditions and consumed *ad libitum* the same dry maintenance diet for at least 3 months prior to and 6 months following castration. All cats were castrated within 48 h of each other. All cats gained weight after castration. Mean BW was 4·67 (sd 0·70) kg at the start of the study and 5·93 (sd 1·38) kg at the end of the study, with individual weight gain ranging 3–53 % at 6 months post-neutering. The pre-conception BW of the queens of each cat was compared with the pre- and post-neutering BW of their offspring. The pre-conception BW of the queens was significantly correlated with the offspring's initial BW (ρ = 0·65, *P* = 0·01), final BW (ρ = 0·67, *P* = 0·01) and percentage BW change (ρ = 0·54, *P* = 0·04). A wide range of post-castration weight gains was observed among cats of similar backgrounds and housing conditions. Implementation of effective methods to control food consumption pre-conception and post-neutering may be a strategy for preventing obesity and obesity-related disorders in cats.

Castration is one factor contributing to the development of obesity in male cats. Hormonal variations^(^^1^^)^ and increased food intake^(^^2^^,^^3^^)^ are associated with body weight (BW) gain following neutering. Other variables influencing weight gain in cats, include age, activity level, housing (indoor or outdoor), diet regimen and feeding schedule (*ad libitum v.* meal feedings)^(^^4^^–^^7^^)^. In human subjects and rodents, maternal obesity at conception has been associated with programming offspring to a predisposition to obesity later in life^(^^8^^,^^9^^)^. However, this association has not been investigated in cats.

The increased prevalence of obesity in cats has concomitantly resulted in greater incidences of obesity-related complications, including diabetes mellitus, dermatological disorders and lameness^(^^10^^)^. Oftentimes, reported incidences of feline obesity following gonadectomy derive from veterinary clinic records^(^^4^^,^^7^^)^, surveys^(^^5^^)^ or prospective studies using small numbers of cats in a controlled environment^(^^1^^–^^3^^)^. In these studies, diverse genetic factors, owner influences, neutering age and environmental/husbandry factors make it difficult to determine individual variations in BW gain due to gonadectomy alone. Furthermore, studies in controlled environments that only report mean (plus standard deviations or standard errors) values for weight gain do not provide information about the per cent of the population showing negligible weight gain or weight gain above a certain percentage of initial BW (i.e. 20 %). Large data sets with individual BW data are needed to obtain a better determination of the distribution of weight gain in response to neutering.

The purpose of the present study was 2-fold. First, the study determined the distribution of weight gain following neutering in a group of weight stable male cats that were maintained under the same environmental conditions before and after neutering. BW change was also followed in a parallel group of age-matched control cats. Second, the study determined if pre-conception BW of queens was correlated with the magnitude of weight gain in their offspring following neutering.

## Materials and methods

### Cats

Twenty-seven specific-pathogen-free young (2·0–2·6 years, median = 2·2 years), intact male domestic shorthaired cats were studied. Cats were group-housed, had *ad libitum* food and water access, and were weight stable for 1·5 months prior to castration. Two groups of cats were studied, a control group of seven intact cats (referred to as cats C1–C7), and another group of twenty that would undergo castration (referred to as cats 1–20). Mean BW at the start of the study was 4·60 (sd 0·64) kg and 4·67 (sd 0·70) kg for the control and castration groups, respectively. Full sibling pairs from the same litter were also identified to determine if genetic similarity promoted uniform post-neutering weight gains between siblings. Among the castration groups, there were three pairs of siblings. The control group also included one sibling pair and one cat with a sibling in the castration group. Control and castrated cats were group-housed by neuter status in 3·5 m × 11 m pens and managed using the same husbandry protocol at the University of California, Davis. The facility maintained room temperatures between 18 and 24°C and a 14·00 h light/10·00 h dark cycle.

The University of California, Davis Institutional Animal Care and Use Committee approved all experimental protocols (Animal Welfare Assurance Number A3433-01).

### Diets

Cats were fed on a dry maintenance diet that was formulated to meet the nutritional recommendations established by the Association of American Feed Control Officials^(^^11^^)^ cat food nutrient profiles for all life stages. This is the standard colony diet and it has been used in previously published studies^(^^12^^)^. The nutrient composition of this diet on a DM basis was 38 % protein, 32 % carbohydrate, 17 % fat, 4 % crude fibre and 9 % ash (determined by the proximate analysis). The metabolisable energy for this diet on a DM basis, calculated using the AAFCO formula^(^^11^^)^, was 16·19 kJ/g (or 3·87 kcal/g). This diet provided 34 % of the energy from protein, 37 % from fat and 29 % from carbohydrates.

### Procedures and data collection

The health of each cat was assessed by physical examination prior to the start of the study. All cats were fed *ad libitum* on the dry maintenance diet for at least 3 months prior to castration. All cats in the castration group were neutered within 48 h of one another using a standard open technique^(^^12^^)^ and allowed *ad libitum* access to food and water for 6 months post-castration. BW are measured weekly for all cats as part of the facility's standard protocols. The primary outcome measure was per cent weight gain from pre-castration to 6 months post-castration. This was a retrospective study, and food intake and body composition measurements were not available for this population of cats. Pre-conception BW from the queens of the castrated cats was retrospectively analysed for associations between maternal weight and offspring weight gain in response to neutering.

### Statistical analysis

All data were tested for normality using the Shapiro–Wilk test prior to statistical analysis (SAS Version 9·2, SAS Institute; Cary, NC). Pearson correlation coefficient (ρ) and its *P* value for significance of correlation were calculated to assess the magnitude and direction of the association between two BW measures (BW measurements in offspring pre- and post-castration, and BW measurements in queens and offspring pre- and post-castration). A two-side *P* value of 0·05 was considered significant. All data are reported as means and standard deviations unless specified otherwise.

## Results

All twenty castrated cats gained weight following neutering. One cat (cat 9) died unexpectedly 2 weeks before the project ended from causes unrelated to the study. At the time of death, this cat's BW had increased by 24 %. The mean BW for the castrated group at the start of the study was 4·70 (sd 0·71) kg which differed from the mean BW, 5·93 (sd 1·38) kg, at the end of the study (*n* 19 cats, *P* < 0·01; Table 1). The increase in BW 6 months after castration ranged from 3 % (cat 11) to 53 % (cats 17 and 18) (mean = 26 (sd 14) %). The greatest number of cats (eight) had BW changes in the range of 11–20 %. In contrast, cats in the control group (*n* 7) started with a mean BW of 4·60 (sd 0·64) kg. After 6 months, they had a mean BW of 4·65 (sd 0·57) kg and a mean percentage change in BW of 2 (sd 5) % (median = 4 %). Pre-neutering and 6 months post-neutering BW did not differ (*P* > 0·10) in the control group.

**Table 1.** Body weight (BW) changes observed in twenty-seven young adult cats, prior to and 6 months after castration. Cats 1–20 were in the castration group. Cats C1–C7 were in the control group

Cat ID	Initial BW (kg)	Final BW (kg)	Change in BW (%)
1	4·60	5·48	19
2	3·85	4·59	19
3	4·69	5·41	15
4	4·58	5·50	20
5	6·60	9·10	38
6	4·41	5·32	21
7	4·27	5·64	32
8	3·85	4·52	18
9*	4·14	–	–
10	4·61	5·17	12
11	3·94	4·08	3
12	4·18	5·18	24
13	5·41	6·15	14
14	4·45	5·10	15
15	4·08	5·07	24
16	4·92	6·10	24
17	4·57	7·01	53
18	5·32	8·12	53
19	5·12	6·49	27
20	5·81	8·62	48
Castration group (means and standard deviations)	4·70 (0·71)	5·93 (1·38)	26 (14)
C1	5·27	5·54	5
C2	4·63	4·80	4
C3	4·40	4·37	−1
C4	4·96	4·70	−5
C5	3·59	3·83	7
C6	4·04	4·25	5
C7	5·31	5·08	−4
Control group (means and standard deviations)	4·60 (0·64)	4·65 (0·57)	2 (5)

*Cat 9 died 2 weeks prior to the end of the study. His last BW measurement was 5·15 kg.

**Fig. 1.**
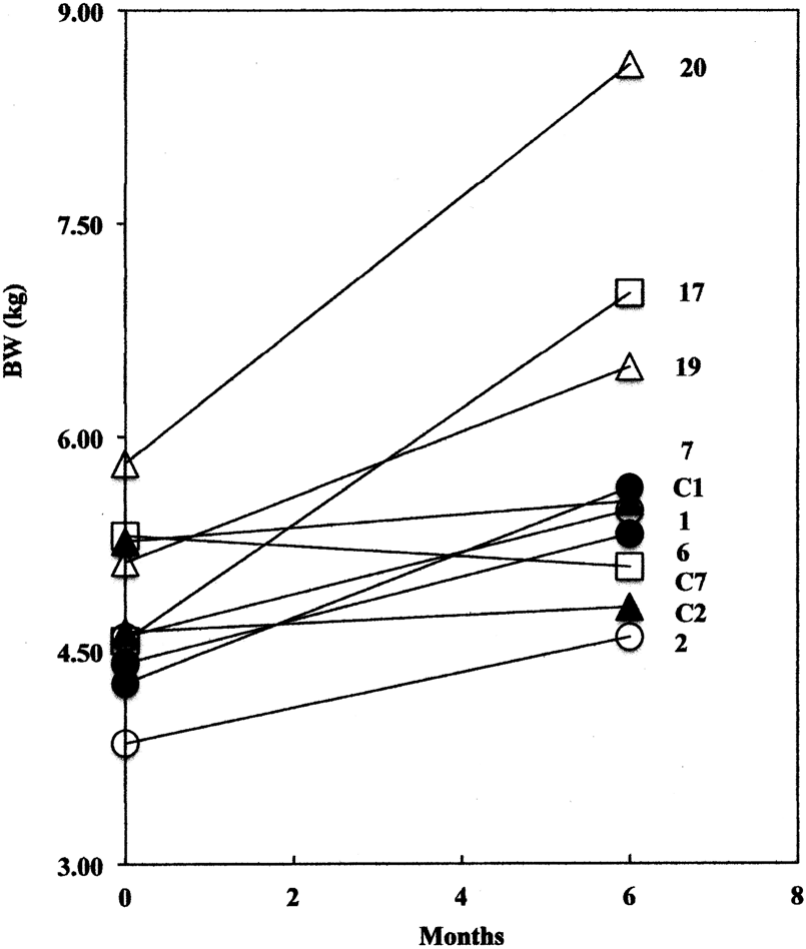
Initial and final body weights (BW) observed in five sibling pairs 6 months after castration. Sibling pairs are cats 1 & 2 (○), 6 & 7 (•), 19 & 20 (∆), C1 & C2 (▲), 17 & C7 (□). Cats C1, C2 and C7 are from the control group.

Weight gain in full sibling pairs was observed to determine if genetic similarity promoted uniform weight gain in siblings (Fig. 1). There was considerable variability in weight gain within sibling pairs with the greatest difference occurring in a pair that showed 48 % (cat 20) *v.* 27 % (cat 19) increases in BW.

Pre-conception BW (mean = 3·47 (sd 0·51) kg) were available for thirteen of the seventeen queens from which the castrated cats' were derived. Retrospective analyses of the weights from the queens showed that prior to conception, the calculated CV for the queens' BW was 0·15. The pre-conception BW of the queens was correlated with the offspring's initial BW prior to castration (ρ = 0·65, *P* = 0·01), the offspring's final BW after castration (ρ = 0·67, *P* = 0·01) and the offspring's percentage BW change at the end of the study (ρ = 0·54, *P* = 0·04). Regression analysis showed that the offspring's initial BW prior to castration was correlated with the offspring's percentage change in BW during the 6 months (*P* = 0·03, *R*^2^ 0·25). However, after adjusting for the queen's BW, this relationship was NS (*P* = 0·09, *R*^2^ 0·33).

## Discussion

Controlled prospective studies on the effects of neutering on BW gain in cats have generally compared weight gain between small numbers (*n* < 10) of intact and castrated animals as a group to determine the mean change in BW, and these studies have not reported individual weight changes which would provide more detailed information about the distribution and range of weight change in response to castration^(^^3^^,^^13^^)^. Frequently, these cats have different backgrounds and vary in age. Thus, the goal of this retrospective study was to evaluate the distribution of weight gain for 6 months following castration in twenty male cats of similar age and background, and in relation to a control group of intact cats. Increased food intake following gonadectomy reportedly contributes to weight gain^(^^2^^,^^3^^,^^13^^)^, and the cats in the present study were provided *ad libitum* access to food. However, food intake and body composition measurements were not available for the animals in this retrospective study.

One study reported the average percentage weight gain in six cats was 17 and 29 % at 1 and 3 months post-castration, respectively, whereas percentage weight gain in the six intact control cats was 10 and 12 % at those same time points^(^^2^^)^. In the present study, the change in BW for the castrated group 1 month after castration ranged from −1 to 17 % (mean = 7 %, median = 6·5 %), with fifteen cats showing <10 % change in weight. Three months post-castration, change in BW ranged from −1 to 43 % (mean = 18 %, median = 14·5 %). Alternatively, cats in the control group showed weight changes of −4 to 2 % after 1 month and −2 to 4 % after 3 months. In contrast to Fettman *et al.*^(^^2^^)^, the cats in the present study were group-housed instead of individually housed, so weight gain may have occurred at a slower rate due to possible increases in physical activity in group-housed animals housed in larger enclosures. Also, the control cats in the Fettman *et al.*^(^^2^^)^ study were not in energy balance, possibly due to changes in housing or husbandry conditions during the study, and the conditions which influenced weight gain in control cats probably also had an impact on BW gain in the castrated cats. Furthermore, the cats in the Fettman *et al.*^(^^2^^)^ study were fed *ad libitum* on Hill's c/d and the relatively low-fibre content in this diet compared with the diet used in the present study may have promoted greater food intake. In another study, the BW change in individually housed, freely fed, normal and lipoprotein lipase-deficient male cats was approximately 28 % greater than pre-neutering weight at 9 months following castration^(^^3^^)^. The weight gain was attributed mainly to a doubling of fat mass^(^^3^^)^. Intact cats exhibited no change in BW and lipoprotein lipase deficiency did not prevent cats from gaining weight post-neutering. These cats were fed on Purina ONE salmon and tuna flavour, which contained less fat and fibre than the diet used in the present study. In another study^(^^1^^)^, group-housed cats with freely available food (composition not given) were monitored for 13 months after neutering to evaluate BW. BW were significantly greater than baseline weights after 8 months, and 20 % greater after 10 months, when weights stabilised.

In the present study, the mean change in BW 6 months after castration in nineteen cats was 26 (sd 14) %. Although comparable, our average value was greater than the Martin *et al.* study^(^^1^^)^ but lower than the Kanchuk *et al.* study^(^^3^^)^. The rate of weight gain reported in the Kanchuk *et al.*^(^^3^^)^ study was likely affected by individual housing. The extended duration it took for cats to reach a stable weight in the Martin *et al.*^(^^1^^)^ study may have been influenced by the age at which the cats were neutered (11 months), since they were likely still growing. At the time the present study ended, the mean BW (5·93 (sd 1·38) kg) of the nineteen cats had not stabilised and was still increasing.

Activity was not monitored in the present study, but since cats were group-housed, they may have exercised more than cats maintained individually in smaller enclosures, and therefore gained weight at a slower rate. Prior to castration, the cats had already been group-housed for at least 2 months with no observed dominance/aggressive interactions or food-guarding behaviours. Previous observations at the facility where the cats were housed showed that castrated male cats usually coexist well together and displays of aggression rarely occur.

In the present study, a positive correlation existed between post-castration BW gain in the cats and the BW of their queens at pre-conception. Studies in human subjects and rodents indicate that maternal obesity may be a factor that predisposes offspring to weight gain later in life^(^^8^^,^^9^^)^. Although BW alone is a crude indicator of obesity, the results of the present study suggest that weight gain in neutered male cats may also be influenced by maternal obesity.

The present study has several limitations, including the fact that food intake, energy expenditure and physical activity were not measured in the cats. Without this information, it is not possible to determine the cause of the variation in weight gain following neutering. Also, body condition scores and body composition information were not available for the queens of the cats used in the study. This information is necessary to truly determine if obesity prior to conception influences future weight gain of the offspring. Future studies focusing on the cats which are either the most resistant or most susceptible to weight gain following neutering are needed to identify factors, which may predict an individual animal's susceptibility to weight gain with neutering.

In conclusion, the present study demonstrated that large variations in post-castration weight gain occur in age-matched adult male cats maintained in the same housing environment. However, all but one cat showed at least a 10 % increase in BW by 6 months post-castration. The predisposition of cats to obesity and other metabolic diseases may be partially programmed at conception. Implementation of effective methods to control food consumption to prevent weight gain at pre-conception and at post-neutering may be a strategy with important implications for preventing obesity and obesity-related disorders in cats.
